# Artifactual FA dimers mimic FAHFA signals in untargeted metabolomics pipelines

**DOI:** 10.1016/j.jlr.2022.100201

**Published:** 2022-03-18

**Authors:** Alisa B. Nelson, Lisa S. Chow, Curtis C. Hughey, Peter A. Crawford, Patrycja Puchalska

**Affiliations:** 1 Division of Molecular Medicine; Department of Medicine, University of Minnesota, Minneapolis, MN, USA; 2 Bioinformatics and Computational Biology Program, University of Minnesota, Minneapolis, MN, USA; 3 Division of Diabetes, Endocrinology and Metabolism; Department of Medicine, University of Minnesota, Minneapolis, MN, USA; 4 Department of Integrative Biology and Physiology, University of Minnesota, Minneapolis, MN, USA; 5 Department of Biochemistry, Molecular Biology, and Biophysics, University of Minnesota, Minneapolis, MN, USA

**Keywords:** insulin resistance, lipidomics, lipids, obesity, adipose tissue, LC-MS/MS, olefinic bond, isobaric FA dimers, lipokines, spectral database, AcN, acetonitrile, AGC, automatic gain control, ^13^C_4_-βOHB, ^13^C_4_-β-hydroxybutyrate, [^13^C_18_] 18:1, ^13^C_18_-Oleic acid, d_8_-Phe, d_8_-phenylalanine, d_8_-Val, d_8_-Valine, FAHFA, FA ester of hydroxy FA, FS, full scan, HESI, heated ESI, HILIC, hydrophilic interaction LC, HPA, hydroxy-palmitic acid, IS, internal standard, OAHPA, oleic acid ester of hydroxy palmitic acid, OAHSA, oleic acid ester of hydroxy stearic acid, PAHPA, palmitic acid ester of palmitic acid, PAHSA, palmitic acid ester of hydroxy stearic acid, RP, reverse phase, RT, retention time

## Abstract

FA esters of hydroxy FAs (FAHFAs) are lipokines with extensive structural and regional isomeric diversity that impact multiple physiological functions, including insulin sensitivity and glucose homeostasis. Because of their low molar abundance, FAHFAs are typically quantified using highly sensitive LC-MS/MS methods. Numerous relevant MS databases house in silico-spectra that allow identification and speciation of FAHFAs. These provisional chemical feature assignments provide a useful starting point but could lead to misidentification. To address this possibility, we analyzed human serum with a commonly applied high-resolution LC-MS untargeted metabolomics platform. We found that many chemical features are putatively assigned to the FAHFA lipid class based on exact mass and fragmentation patterns matching spectral databases. Careful validation using authentic standards revealed that many investigated signals provisionally assigned as FAHFAs are in fact FA dimers formed in the LC-MS pipeline. These isobaric FA dimers differ structurally only by the presence of an olefinic bond. Furthermore, stable isotope-labeled oleic acid spiked into human serum at subphysiological concentrations showed concentration-dependent formation of a diverse repertoire of FA dimers that analytically mimicked FAHFAs. Conversely, validated FAHFA species did not form spontaneously in the LC-MS pipeline. Together, these findings underscore that FAHFAs are endogenous lipid species.  However, nonbiological FA dimers forming in the setting of high concentrations of FFAs can be misidentified as FAHFAs. Based on these results, we assembled a FA dimer database to identify nonbiological FA dimers in untargeted metabolomics datasets.

## Graphical abstract

**Figure:**
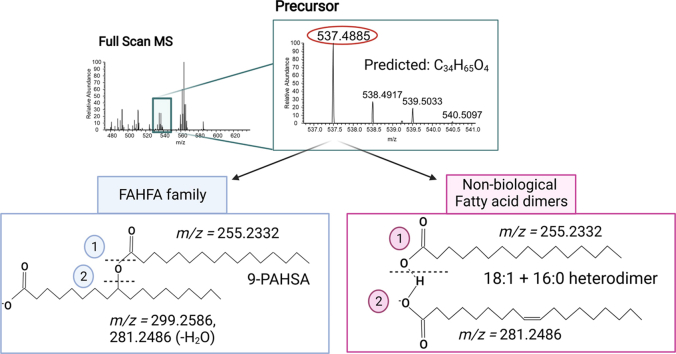

Metabolomics is the study of global changes of the metabolome in biological samples and has risen to prominence, especially in human studies, to detect possible biomarkers of disease onset and progression (1, 2, 3, 4, 5). Metabolomics consists of targeted and untargeted approaches for quantitative and semiquantitative acquisition of metabolite abundances, respectively. Untargeted methods are designed to maximize coverage of the metabolome and discover perturbed metabolites, whereas targeted approaches use extraction protocols and acquisition methods that have been optimized for specific metabolic compounds and/or classes (6, 7, 8, 9). Untargeted metabolomics, while useful for casting a wide net to survey disrupted metabolites, requires substantial effort dedicated to data analysis to *i*) remove nonbiologically relevant signals, such as background ions, adducts, fragment ions, and isotopes and *ii*) validate the identities of features of interest (10, 11). Because of instrument sensitivity and ionization technology, adducts and fragment ions are features of metabolomics pipelines that can confound researchers as they can form reproducibly and contain identifiable fragments (12, 13). For this reason, validation of putative metabolites with biological impact, by fragmentation, authentic internal standard (IS), and their formal quantitation using targeted approaches, is an important further stage in untargeted metabolomics studies (14).

FA esters of hydroxy FAs (FAHFAs) first identified by Yore *et al.* (15) have been linked to insulin sensitization and are composed of two FAs linked via a hydroxyl ester group. Palmitic acid ester of hydroxy stearic acid (PAHSA), the most studied FAHFA species, is of interest for its role in enhancing insulin sensitivity. Treatment with 5-PAHSA and 9-PAHSA in mice fed a high fat diet improved glucose uptake and insulin sensitivity in heart, skeletal muscle, and liver (16). Elucidation of underlying mechanisms of FAHFA impact on glucose homeostasis remains ongoing and requires robust measurement methods to quantify a diverse repertoire of FAHFA species, including their regioisomers (e.g., 5-PAHSA vs. 9-PAHSA), in both human and nonhuman biospecimens. FAHFAs are present at low nanomolar concentrations, similar to other signaling lipids such as prostaglandins (15). Thus, optimized quantitation of FAHFAs requires extraction of lipids, followed by solid-phase extraction and separation through high-performance LC system (15, 17). Other methods employ derivatization to increase sensitivity and to separate FAHFAs from confounding signals, such as ceramides in electrospray probe (18, 19). Thus, appropriate measurements of changes to FAHFA concentrations should be performed in targeted experiments rather than using semiquantitative and untargeted methods.

We performed an untargeted metabolomics experiment using a commonly applied hydrophilic interaction LC coupled to ESI MS (HILIC-ESI-MS) method (20, 21, 22, 23, 24, 25, 26) and observed that multiple signals matching *m/z* of several FAHFAs were detected in human serum extracts. MS/MS databases putatively identified multiple FAHFA signals in studied human serum such as PAHSA, as well as oleic acid ester of hydroxy stearic acid (OAHSA), and palmitic acid ester of palmitic acid (PAHPA). Further validation studies, including the use of authentic standards, confirmed that the species detected in this MS pipeline were not in fact FAHFAs but artifacts formed from two interacting FFAs in the mass spectrometer.

## Materials and methods

### Chemicals and materials

LC-MS grade methanol (MeOH), acetonitrile (AcN), and water were purchased from Thermo Fisher Scientific. Ammonium acetate and formic acid were purchased from Honeywell. ISs d_8_-phenylalanine (d_8_-Phe), d_8_-valine (d_8_-Val), ^13^C_4_-β-hydroxybutyrate (^13^C_4_-βOHB), and ^13^C_18_-Oleic acid ([^13^C_18_] 18:1) were purchased from Cambridge Isotopes. 9-PAHSA, d_4_-PAHSA (9-PAHSA-d_4_), OAHSA (5-OAHSA and 12-OAHSA), and d_17_-OAHSA (5-OAHSA-d_17_) were purchased from Cayman Chemical. Human serum used for [^13^C_18_] 18:1 validation experiment was acquired from Sigma-Aldrich.

### Human serum sample collection

Normal weight trained (*n* =14) participants from Minneapolis or St. Paul, MN, USA were recruited between July 2014 and April 2017. The exercise bout was a prolonged run designed to promote fat oxidation. Subjects were instructed to avoid intentional exercise for 2 days before the second visit and arrive after an overnight fast (at least 8 h). For the supervised exercise bout, all subjects ran for 90 min on a treadmill. Subjects were offered free access to water during their exercise bout. Plasma samples were collected before and after the acute aerobic exercise intervention. The University of Minnesota’s Institutional Review Board approved the study protocol and methods. All participants provided written informed consent before study participation. The study abides by the Declaration of Helsinki principles.

### Sample preparation for untargeted metabolomics study

Samples were stored at −80°C and extracted just prior to the analysis using the protocol from Ivanisevic *et al.* (21) with modifications (21, 25, 26). For quality control, serum was spiked with ISs of d_8_-Phe, d_8_-Val, and ^13^C_4_-βOHB (10 μM each in final extract), normally absent in the serum and ion intensity monitored along the batch analysis. Briefly, 20 μl of serum was extracted with 80 μl of cold (−20°C) MeOH:AcN (1:1, v/v) solution and submitted to the vortexing and bath sonication (10 min). The samples were incubated at −20°C for 1 h, spun to remove proteins, transferred to fresh tube, evaporated, and reconstituted in 100 μl of 1:1 AcN:H_2_O (1:1, v/v). Prepared samples were vortexed, sonicated (10 min), centrifuged (10 min, 13,000 rpm at 20°C), and analyzed.

### Untargeted LC-MS metabolomics analysis

Untargeted analysis was performed on Thermo Vanquish liquid chromatograph hyphenated with Thermo Q-Exactive Plus mass spectrometer equipped with heated ESI (HESI) source. Samples were analyzed by HILIC separation conditions, using negative mode on Luna Aminopropyl (150 × 1 mm, 3 μm) using mobile phase A, 10 mM ammonium acetate/10 mM ammonium hydroxide in 95% water, and mobile phase B, 10 mM ammonium acetate/10 mM ammonium hydroxide in 95% AcN. Separation was performed using binary gradient of 100–0% B for 50 min, 0% B for 7 min, and 100% B for 13 min. Separations were performed at a flow rate 50 μl/min and column temperature at 30°C with an injection volume of 2 μl. The mass spectrometer operated in negative full scan (FS) mode (*m/z* 68–1,020) was used with optimized HESI source conditions: auxiliary gas 10, sweep gas 1, sheath gas flow at 35 (arbitrary unit), spray voltage −3 kV, capillary temperature 275°C, S-lens RF 50, and auxiliary gas temperature 150°C. The automatic gain control (AGC) target was set at 1e6 ions and resolution at 70,000. Samples within the sequence were injected in randomized order to minimize the possibility of column carryover. The signals of d_8_-Phe, d_8_-Val, and ^13^C_4_-βOHB were extracted from all analyzed samples, and the relative standard deviation of the area among all samples was below acceptable 10%. Features of interest were targeted by parallel reaction monitoring using an *m/z* 1.0 window and 5-min retention time (RT) window for each signal. For targeted MS/MS of ions, the following MS/MS settings were applied: resolution at 35,000, AGC target of 2e5, and maximum injection time of 100 ms. Normalized collision energy was applied in steps of 20, 40, and 100%.

### Unknown lipid identification study

Studies were performed using the HILIC-ESI(−)-MS/MS method described previously using a Luna Aminopropyl (100 × 1 mm, 3 μm) column, and reverse-phase (RP) LC-MS (RP-(ESI−)-MS/MS) was performed using Thermo Hypersil Gold C18 column (150 × 1 mm, 3 μm) in negative ionization mode. Separation conditions for RP-(ESI−)-MS/MS were performed as previously described, with modifications (27). Briefly, mobile phase A contained 10 mM ammonium formate in 60/40 (v/v) AcN/H_2_O with 0.1% formic acid. Mobile phase B contained 10 mM ammonium formate in 90/10 (v/v) isopropanol/AcN with 0.1% formic acid. Separation used a binary gradient of 5% B for 3 min, 5–15% B for 2 min, 15–48% B for 5 min, 48–82% B for 5 min, 82–95% B for 3 min, 95–15% B for 5 min, 15–5% B for 4 min, and 5% B for 3 min. Separations were performed with a flow rate of 150 μl/min and column temperature of 45°C with injection volume of 2 μl. The mass spectrometer operated in negative ionization and FS mode (mass range *m/z* 220–1,700) with parallel reaction monitoring of precursor ions: *m/z* = 580.6112 (5-OAHSA-d_17_), 563.5045 (detected unknown lipid C36:1 and OAHSA), 537.4888 (detected unknown lipid C34:0 and PAHSA) and used optimized HESI source conditions: auxiliary gas 10, sweep gas 1, sheath gas flow at 35 (arbitrary unit), spray voltage −3 kV, capillary temperature 275°C, S-lens RF 50, and auxiliary gas temperature 150°C. The AGC target was set at 3e6 ions, and resolution was set at 70,000.

Exercise serum samples were spiked with 5-OAHSA-d_17_ (Cayman Chemical), extracted, and the data were acquired using parallel reaction monitoring to target desired *m/z*. In a separate extraction, serum samples were spiked with 1 μM 5-OAHSA and 20 μM 5-OAHSA-d_17_ to evaluate changes of ion counts of detected *m/z* 563.5045.

### Dimer validation study

Dimer formation experiments used [U-^13^C_18_] 18:1 spiked into human serum (Sigma-Aldrich) at 0.1–50 μM concentration interval. Predicted *m/z* for [U-^13^C_18_] 18:1-containing dimers were targeted for MS/MS. Quan browser was used to integrate detected peaks in FS. Percent of dimer to [U-^13^C_18_] 18:1 signal determined efficiency.

### FAHFA validation study

To confirm FAHFA species as endogenous metabolites rather than artifacts of metabolite extraction or data acquisition, we added 50 μM hydroxy-palmitic acid (HPA). HPA was first conjugated to FA-free BSA to mimic serum conditions as previously described (28, 29). Briefly, Krebs-Henseleit bicarbonate solution (118 mM NaCl, 25 mM NAHCO_3_, 4.7 mM KCl, 1.2 mM KH_2_PO_4_, 2.5 mM CaCl_2_, 1.2 mM MgSO_4_, pH 7.4) was heated to 40–44°C. FA-free BSA was dissolved in Krebs for final concentration of 0.13 mM. About 20 mM HPA was dissolved in ethanol and water. Dissolved HPA was slowly added to BSA and then dialyzed against Krebs-Henseleit solution with a 7 kDa cassette overnight. HPA-BSA was spiked into human serum and extracted for untargeted metabolomics, and data were acquired on HILIC-ESI(−)-MS/MS as described previously.

Direct infusion experiment was performed using MS parameters described previously. About 50 μM [U-^13^C_18_] 18:1 and 50 μM HPA in AcN:H_2_O (1:1) were infused into Thermo QE Plus at a flow rate of 5 μl/min. FS data were acquired in a mass window of *m/z* 200–700 for 5 min. Dimer *m/z* were predicted based on chemical formula and targeted for MS/MS (±0.5 *m/z*) at normalized collision energy = 35, including *m/z* = 543.5, 553.5, 571.5, and 599.6. Fragmentation data were acquired for 3 min.

### Mouse study

Serum was obtained from submandibular bleed of 8-week-old wild-type male mice from the C57BL/6NJ background (*n* = 6; Jackson Laboratory) after a 6-h fast that began within 2 h after the start of light cycle, as previously described (8). Serum extracted as described previously for detection of dimers. All animal experiments were approved by the Institutional Animal Care and Use Committee at the University of Minnesota.

## Results

### Detected lipid signals resemble FAHFAs in untargeted LC-MS pipelines

**Fig. 1:**
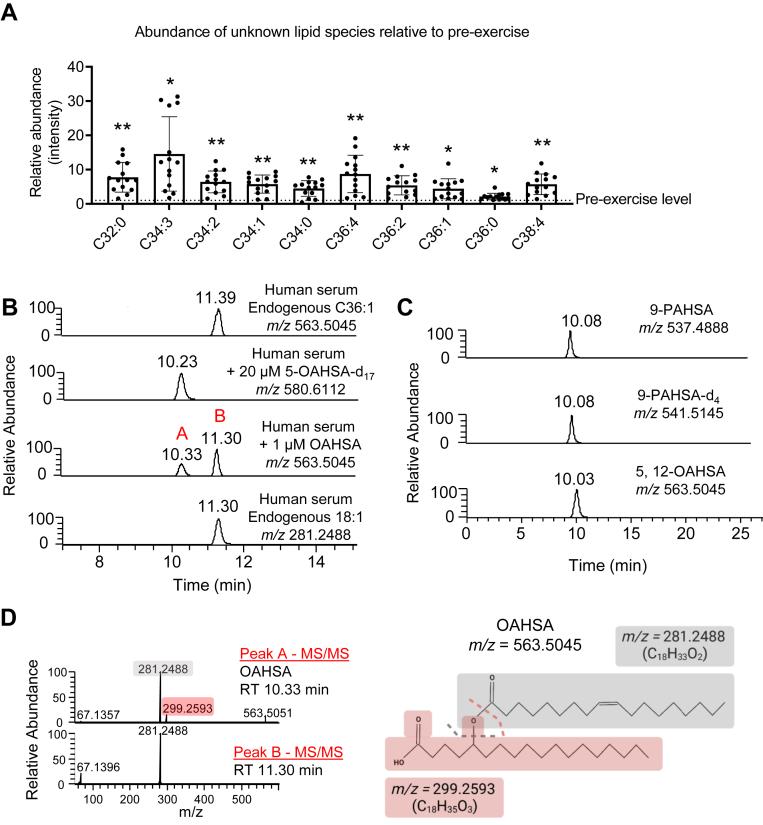
Detected lipid signals resemble FAHFAs in untargeted LC-MS pipelines. A: Fold change of detected unknown lipid species relative to pre-exercise. Naming of the lipid species determined by predicted chemical formula. ∗*P* ≤ 0.001; ∗∗*P* ≤ 0.0001 by Student’s *t*-test with Benjamini-Hochberg for multiple testing. Data represent SEM. B: XIC (±5 ppm) corresponding to *m/z* = 563.5045 in human serum, *m/z* = 580.6112 in human serum spiked with 20 μM labeled 5-OAHSA-d17, *m/z* = 563.5045 in human serum spiked with 1 μM exogenous 12-OAHSA, or *m/z* = 281.2486 (endogenous FFA 18:1) in human serum. C: XICs (±5 ppm) of authentic FAHFA regioisomers dissolved in AcN/H_2_O. D: Schema of reported OAHSA fragmentation pattern and MS/MS spectra of peak A and peak B (from XIC shown in (B)). XIC, extraction ion chromatogram.

Serum collected from lean healthy participants before and immediately after a 90-min treadmill run was analyzed using a hydrophilic high-performance HILIC system hyphenated with high-resolution and high mass accuracy MS operated in negative mode (ESI(−)-MS) using FS mode (26). Metabolite extraction and HILIC-ESI(−)-MS method were optimized for coverage of the metabolome (21). Using a standardized metabolomics pipeline, differential analysis revealed numerous independent chemical features that increased 5-fold to 15-fold with acute aerobic exercise (Fig. 1A, supplemental Table S1). Similarities among their *m/z*, RTs, and targeted MS/MS fragmentation patterns suggested they were all species derived from the same lipid class (supplemental Fig. S1). A subset of the same signals was also detectable in extracts of mouse serum, collected after a 6 h fast, and analyzed using the same metabolomics pipeline (supplemental Fig. S2A). Database searches based on *m/z* and fragmentation patterns indicated that several of these features putatively matched known species from the FAHFA lipid class (supplemental Table S2). However, spiking a deuterated FAHFA authentic standard 5-OAHSA-d_17_ into human serum revealed that the interrogated putative endogenous lipid species signal we observed did not coelute with spiked FAHFA standards (Figs. 1B and S2B). Importantly, the RT of these isobaric signals showed a distinct time shift of only 1 min. Spiking unlabeled 5-OAHSA into postexercise serum showed the coelution of spiked and labeled OAHSA signals, and the appearance of two unlabeled chromatographic signals (*m/z* = 563.5045) (Fig. 1B). This suggested that the endogenous signals of interest were different from FAHFAs, denoted as peak A (OAHSA) and peak B (unknown). Since FAHFAs can be further distinguished by the ester bond site using RP chromatographic separation (15), we determined if peak A and peak B were regioisomers of FAHFAs. A standard sample composed of 9-PAHSA, 9-PAHSA-d_4_, 5-OAHSA, and 12-OAHSA showed that various isomers of FAHFAs coeluted using our HILIC chromatographic method (Fig. 1C). These findings suggest that HILIC chromatography does not separate FAHFA regioisomers, thus peak B is likely a chemical entity distinct from any FAHFA family member. Interrogation of the fragmentation pattern of each *m/z* = 563.5045 signal showed they are highly similar but not identical (Fig. 1D). Peak A (RT = 10.33 min) identified as OAHSA showed two main fragments, *m/z* = 299.2593 (corresponding to hydroxy stearic acid) and *m/z* = 281.2488 (corresponding to oleic acid). These fragments match reported qualitative fragments for OAHSA identification (15, 30). The disputed peak B (RT = 11.30 min) contains only one fragment corresponding to oleic acid with *m/z* = 281.2488. Both peak A and peak B release a fragment of *m/z* = 67.13; however, this could not be matched to any predicted chemical formula. The absence of the hydroxy FA fragment in the MS/MS spectra of feature *m/z* = 563.5045 suggested that the FA chains in the signal of interest are not covalently bound by an ester bond. Interestingly, careful inspection of the extraction ion chromatogram near the RT of peak B showed that FFAs coelute with the unknown lipid features (Fig. 1B). Injection of human serum with spiked deuterated FAHFA authentic standard 5-OAHSA-d_17_ on an RP (C18) column showed separation of the detected C36:1 peak by more than 5 min from FAHFA standards and again the coelution of the signal of interest with FFA 18:1, similar to HILIC-ESI(−)-MS (supplemental Fig. S3A and Fig. 1B). Authentic regional isomers of FAHFA standards of 12-OAHSA, 5-OAHSA, and 9-PAHSA separated in the expected descending order on RP chromatography by location of the ester bond eluting from 14.8-15.3 min, however none of the signals eluted close to the queried investigated signal (9.3–9.4 min) confirming the data obtained from HILIC runs (supplemental Fig. S3A, B) (15, 17). Taken together, these observations indicate that the lipid species mapping to the FAHFA lipid class by MS/MS spectral analysis of serum extracts using our untargeted LC-MS metabolomics pipeline instead belonged to a distinct non-FAHFA lipid class.

### FA dimers form artifactually in LC-MS untargeted metabolomics pipelines

**Fig. 2:**
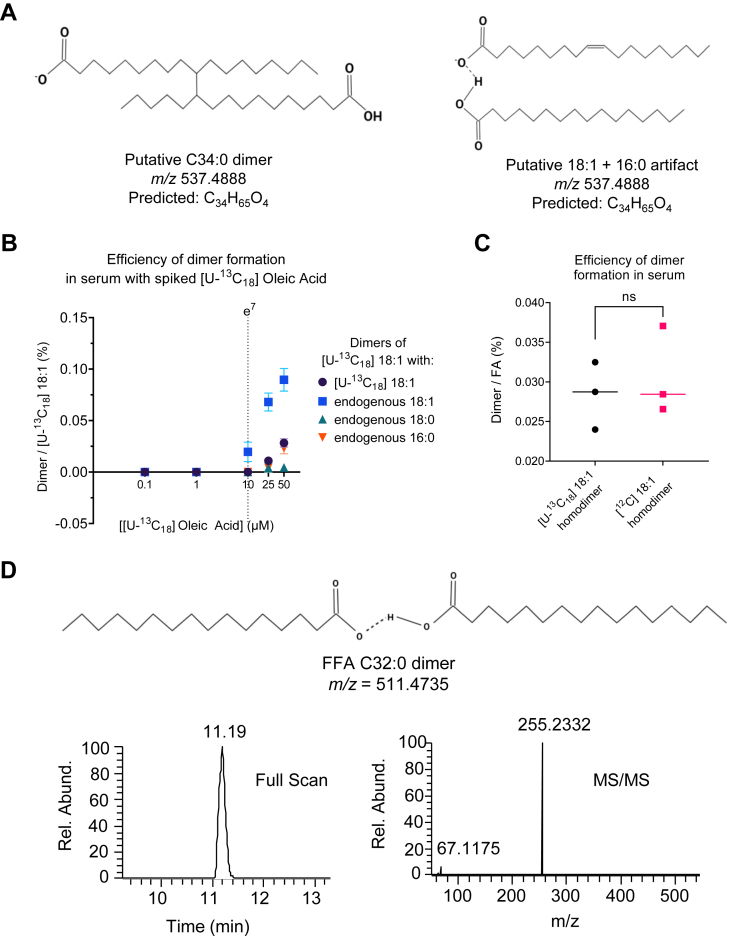
FA dimers form artifactually in LC-MS untargeted pipelines. A: Proposed chemical structure of covalently or noncovalently derived unknown lipid C34:0 based on precursor ion (*m/z*) and predicted chemical formula. B: Efficiency of various nonbiological dimers formed from increasing concentrations of [U-^13^C_18_] 18:1 in human serum with itself (*m/*z = 599.6245) or endogenous FFA 18:1 (*m/z* = 581.5647), FFA 18:0 (*m/z* = 583.5799), or FFA 16:0 (*m/z* = 555.5486). C: Efficiency of forming labeled and unlabeled 18:1 homodimers at equimolar concentrations. D: Proposed chemical structure, XIC (±5 ppm), and MS/MS spectra of a C32:0 dimer. NS, not significant; XIC, extraction ion chromatogram.

Fragmentation patterns of the unknown lipid molecular features indicated the presence of two FA chains, but no distinguishing lipid head group was detected (supplemental Fig. S1). Considering the lipid species of interest are not FAHFAs, but given their same *m/z* as FAHFAs, similar MS/MS fragmentation and co-elution with FFAs, we hypothesized that these features were either (i) endogenous dimers of FAs cross-linked by simple covalent bonds, or (ii) two FA moieties bound through noncovalent interactions (Fig. 2A). Indeed, FFA 18:1 and putative biologically relevant FA dimers (FA dimers) with the same *m/z* as PAHSA (*m/z* = 537.5) and OAHSA (*m/z* = 563.5), were detected by touch spray MS and desorption electrospray ionization MS and reported to increase in tumors (31, 32). Additionally, complex lipid dimers form in rat livers treated with carbon tetrachloride were identified by gas chromatography-MS (33). To determine if the chemical features observed in our experiments are endogenous, and whether they are covalently linked FA dimers, or noncovalent FA dimers forming within the analytical pipeline, uniformly labeled oleic acid ([U-^13^C_18_] 18:1), a signal naturally absent in serum, was spiked into human serum at increasing concentrations. We observed a strong relationship between the added concentration of [U-^13^C_18_]18:1 in serum and signals corresponding to co-eluting [U-^13^C_18_]18:1-derived homodimers (dark blue circles), [U-^13^C_18_]18:1-18:1 heterodimers (light blue squares), [U-^13^C_18_]18:1-18:0 heterodimers (green triangles), and [U-^13^C_18_]18:1-16:0 heterodimers (orange triangles) (Fig. 2B). Importantly, dimer formation occurred when FFA ion counts exceeded an intensity of e^7^. To determine if the detected endogenous 18:1 dimer may also be artifactually formed, we compared the efficiency of [U-^13^C_18_]18:1 homodimer and putative homodimer containing endogenous FFA 18:1 at equimolar concentrations. These showed no significant differences (Fig. 2C). Interestingly, the formation of dimers was not exclusively associated with unsaturated FAs, which may be more amenable to reactions at the double bond. Instead, we also observed the formation of fully saturated FA dimers, including 16:0 homodimer (Fig. 2D). The composition of the 16:0 homodimer was evaluated through MS/MS fragmentation where only a palmitic acid fragment was detected from the precursor, *m/z* = 511.4735. While the 16:0 homodimer does not correspond to any isobaric FAHFA species, artifactually created isobaric FA dimers differ from FAHFAs by the presence of an olefinic bond, by which they can be distinguished using tandem MS (supplemental Table S3). These data suggest that the observed putative FA dimer signal could reflect an artifact formed when FA ions are abundant in the mass spectrometer. Indeed, given that the queried lipid species were markedly increased in abundance immediately following acute aerobic exercise (Fig. 1A), we formally quantified FFA using a targeted shotgun lipidomics approach (26, 34). As expected, numerous FFAs were elevated post-exercise, similar to observed FA dimers. Given that the formation of signals corresponding to FA dimers was observed in direct relation to the concentrations of exogenously added FFAs (Fig. 2B), increasing abundance of these dimeric FA signals is likely explained by the higher abundance of endogenous FFAs immediately following acute exercise in human serum samples. Finally, these FA dimers were confirmed not to be FAHFA lipids by shotgun lipidomics analysis of circulating FAHFAs, most of which decreased following acute aerobic exercise (18, 26).

### FAHFAs are not artifactually formed in LC-MS untargeted metabolomics pipelines

**Fig. 3:**
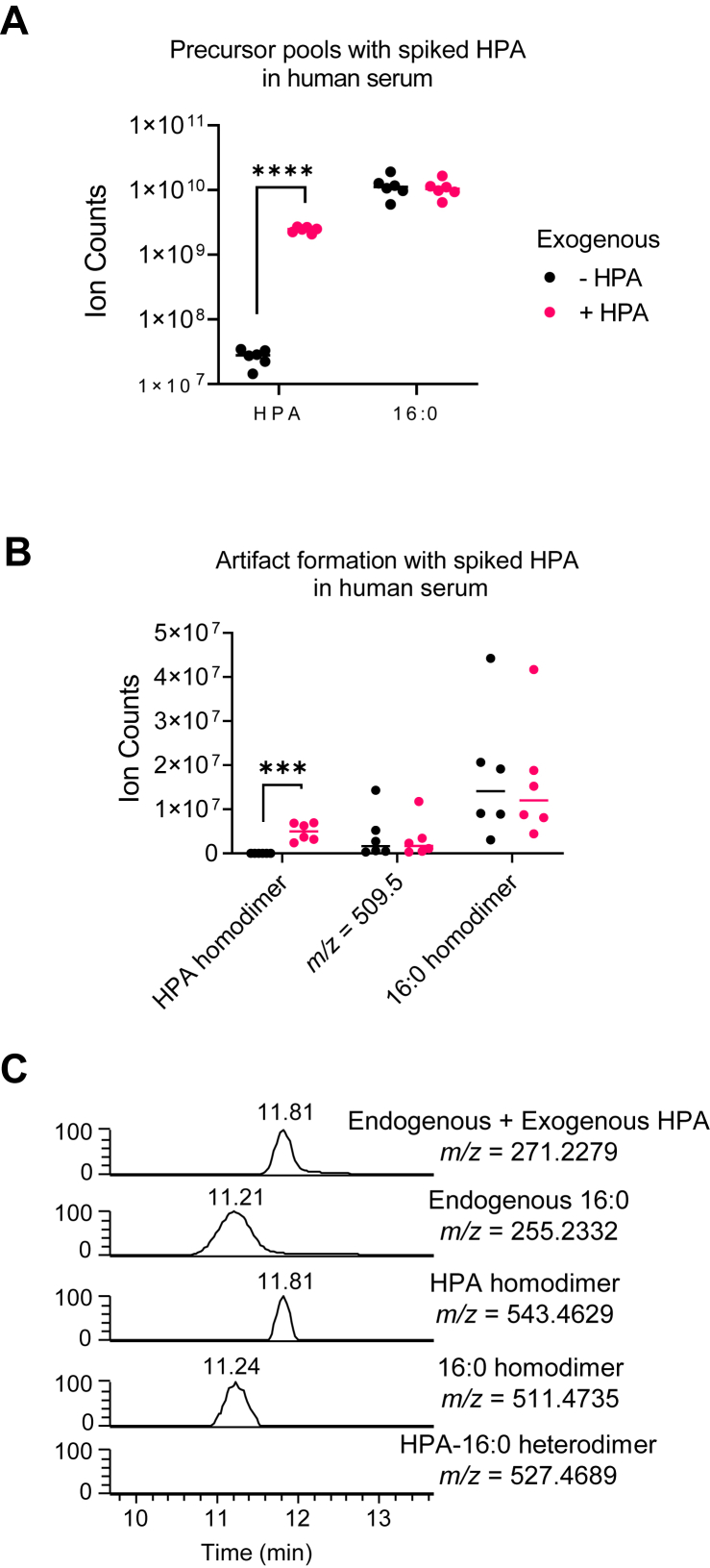
FAHFAs do not form artifactually in untargeted LC-MS pipelines. A: Total ion counts of HPA and palmitic acid (16:0) preaddition (black) and postaddition (magenta) of exogenous HPA. B: Total ion counts of detected HPA homodimer, *m/z* of detected 16:1–16:0 FA dimer (*m/z* = 509.4575, detected at RT = 11.5 min), and 16:0 homodimer preaddition and postaddition of exogenous HPA. Artifactual isobaric PAHPA (*m/z* = 509.4575), not detected at RT = 10.3 min. ∗∗∗*P* ≤ 0.001; ∗∗∗∗*P* ≤ 0.0001 by Student’s *t*-test. Data represent SEM. C: XICs (±5 ppm) of endogenous + 50 μM spiked HPA, endogenous FFA 16:0, HPA homodimer, 16:0 homodimer, and predicted *m/z* of HPA-16:0 heterodimer (undetected). XIC, extraction ion chromatogram.

Our data indicate that at least a subset of detected signals mapping to FAHFAs are in fact FA dimers formed artifactually in hydrophilic and RP LC-MS untargeted analytical pipelines. FAHFAs are composed of one FA chain and one hydroxy FA chain with *m/z* ranges that match detected FA dimers. To ensure that our metabolomics workflow does not also spontaneously form FAHFAs, we spiked serum samples with exogenous hydroxy palmitic acid (HPA) and investigated total ion count of *m/z* = 509.4575 (16:0 + HPA) that would correspond to palmitic acid ester of hydroxy palmitic acid (PAHPA) (Fig. 3A, B). Spiking HPA in human serum samples significantly increased the total pool of endogenous HPA, increasing the total ion count to e^9^, which was sufficient to form HPA homodimer with *m/z* = 543.4636 (Fig. 3A, B). However, this did not impact the pool size of detected *m/z* = 509.4575 ± 5 ppm, suggesting 16:0 and HPA do not form PAHPA in the LC-MS pipeline. At the same time, the intensity of the signal corresponding to FFA 16:0 and nonbiologically formed 16:0-16:0 homodimer was unchanged upon addition of HPA, suggesting HPA neither reacts with 16:0 nor depletes the FFA 16:0 pool. In addition, noncovalent 16:0-HPA heterodimers (*m/z=* 527.4689) were not detected in our LC-MS pipeline (Fig. 3C).

**Fig. 4:**
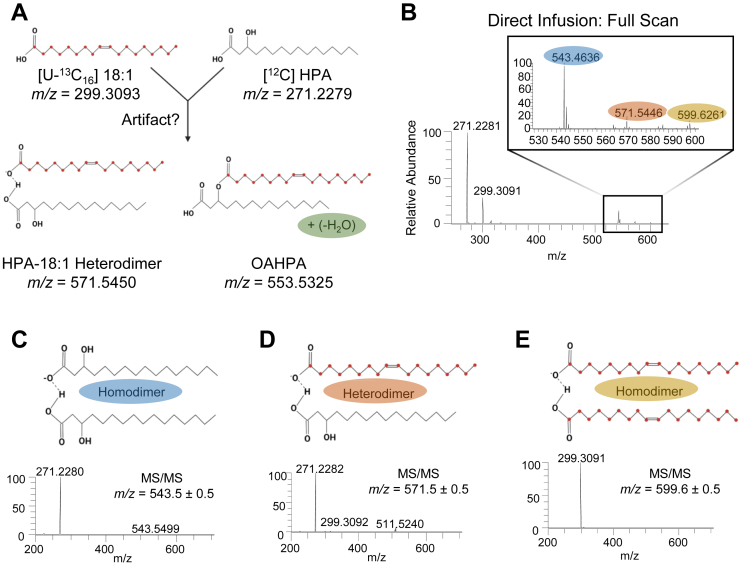
FAHFAs do not form in electrospray with direct infusion. A: Proposed labeling schema of putative artifactually formed HPA-18:1 noncovalent heterodimer and putative artifactually formed OAHPA ([^13^C_18_] 18:1-[O-16:0]). Green label indicates loss of water required to form artifactual OAHPA from reactants. B: FS (MS1) spectra of directly infused solution containing 50 μM [U-^13^C_18_] 18:1 and 50 μM HPA in AcN:H_2_O (1:1). Enlarged: dimer-containing mass range *m/z* 520–610 with corresponding MS/MS spectra of detected (C) HPA homodimer, (D) HPA-18:1 heterodimer, and (E) 18:1 homodimer. Artifactual OAHPA with *m/z* = 553.5325 was not detected.

We next considered that the 16:0-HPA heterodimer might not form because of the separation of 16:0 and HPA molecules in the LC under the conditions we used, HPA eluted at 11.8 min, more than 30 s after 16:0. To determine if FAHFAs are covalently formed when hydroxy FAs and FFAs are together in the mass spectrometer, we prepared a solution of HPA and [U-^13^C_18_] 18:1 for LC-independent direct infusion and investigated *m/z* ranges corresponding to 18:1-HPA heterodimer and [^13^C]-labeled oleic acid ester of hydroxy palmitic acid (Fig. 4A). FSs showed expected *m/z* ratios in the 500–600 mass range corresponding to HPA homodimer (*m/z* = 543.4636), the heterodimer formed between 18:1 and HPA (*m/z* = 571.5446), and 18:1 homodimer (*m/z* = 599.6261) (Fig. 4B–E). MS/MS of the HPA homodimer shows only the HPA product ion (Fig. 4C). Fragmentation of detected HPA-18:1 heterodimer showed product ions corresponding to HPA and [U-^13^C_18_] 18:1 (Fig. 4D). An additional fragmentation signal was observed on this spectrum, *m/z* = 511.5240, which likely corresponds to neutral loss of acetic acid from the HPA. Finally, MS/MS of the [U-^13^C_18_] 18:1 homodimer showed only the [U-^13^C_18_] 18:1 product ion (Fig. 4E). Thus, despite the simultaneous presence of both HPA and 18:1 in the ion source, no signal was detected for an artifactually formed oleic acid ester of hydroxy palmitic acid (predicted *m/z* = 553.5325). Together, these data indicate that unlike FA noncovalent dimers, FAHFAs were not artifactually formed in this commonly applied LC-MS metabolomics pipeline, even when higher HPA concentrations are present in the serum sample.

## Discussion

Untargeted metabolomics reveals metabolite signatures independent of predetermined pathways and is often used to detect metabolites that differ in healthy versus diseased contexts. Detection of differentially abundant metabolites is useful *i*) for directing further mechanistic studies or *ii*) to initiate new approaches for diagnosis and staging disease progression. Here, we revealed the formation of FAHFA-mimicking FA dimers within a commonly used LC-MS untargeted metabolomics pipeline (20, 21, 22, 23, 24, 25, 26) in both human and mouse serum samples on HILIC and RP chromatography. Follow-up studies confirmed that *i*) these FA dimer species were indeed formed in the analytical pipeline, *ii*) they were not FAHFAs, and *iii*) FAHFAs do not form artifactually in the analytical pipeline.

MS-based untargeted metabolomics workflows have been highlighted to show that as high as 90% of the acquired signals are attributable to adducts, contaminants, or artifacts (10, 12). In response, diverse approaches to remove MS artifacts have been developed and deployed, including computational elimination of the background signals, utilization of stable isotopes in the media, addition of labeled isotopes in the mobile phases, annotation of in-source fragments, and expansion of tandem MS databases based on the real standards (11, 35, 36, 37, 38, 39, 40, 41, 42, 43, 44, 45, 46). This effort has significantly improved data analysis, compound identification, and validation in complex biological matrices. Nonetheless, in our studies, FA dimers were preliminarily misidentified as FAHFAs using MS/MS spectra and widely available databases. Indeed, reports of newly observed spectral artifacts observed in high-resolution high mass accuracy mass spectrometers or in-source conversion of highly similar molecules are highlighting outstanding need to validate signals of interest detected in untargeted metabolomics pipelines (13). In our studies, use of authentic standards and stable isotope labeling experiments revealed FA dimer signals to be of nonbiological origin and a result of the interaction of coeluting FAs. These noncovalent interactions could be mediated by charge-charge, charge-dipole, dipole-dipole, charge-induced dipole, dipole-induced dipole, or hydrogen bonding (10). To help differentiate nonbiological FA dimers from FAHFA, we created an in silico database of predicted precursor *m/z* with corresponding fragments to help identify dimers that may form when FAs are abundant in the mass spectrometer (supplemental Table S3).

Circulating FAHFA concentrations correlate directly with insulin sensitivity and may be anti-inflammatory. Moreover, treatment of mice maintained on a high fat diet with the FAHFA species PAHSA improved insulin sensitivity and glucose homeostasis (15, 16, 47). While research has commonly focused on the PAHSA species, in silico studies estimate that more than 1,000 distinct FAHFAs could exist, including both structural and regional isomers, with 20 FAHFA sub-families already known in mammalian tissues (17, 48, 49). The potential impact of FAHFAs on insulin resistance and type 2 diabetes alone makes them an important lipid class for both mechanistic and discovery studies. However, their formal quantitation requires specific extraction protocols and many authentic standards. Thus, for screening protocols, it is important to recognize the presence of confounding signals in biological matrices with high free fatty acid content.

## Data availability

All data are contained within the article.

## Supplemental data

This article contains supplemental data (48).

## Conflict of interest

P. A. C. has served as an external consultant for Pfizer, Inc, Abbott Laboratories, and Janssen Research & Development. All other authors declare that they have no conflicts of interest with the contents of this article.
