# Supplementary material for: Artifactual FA dimers mimic FAHFA signals in untargeted metabolomics pipelines
Source: J Lipid Res. 2022 Mar 18;63(5):100201. doi: 10.1016/j.jlr.2022.100201 (PMC9034316; doi:10.1016/j.jlr.2022.100201)
Supplement: Supplemental Figures S1–S3 [file mmc4.pdf]

Supplemental Figure 1

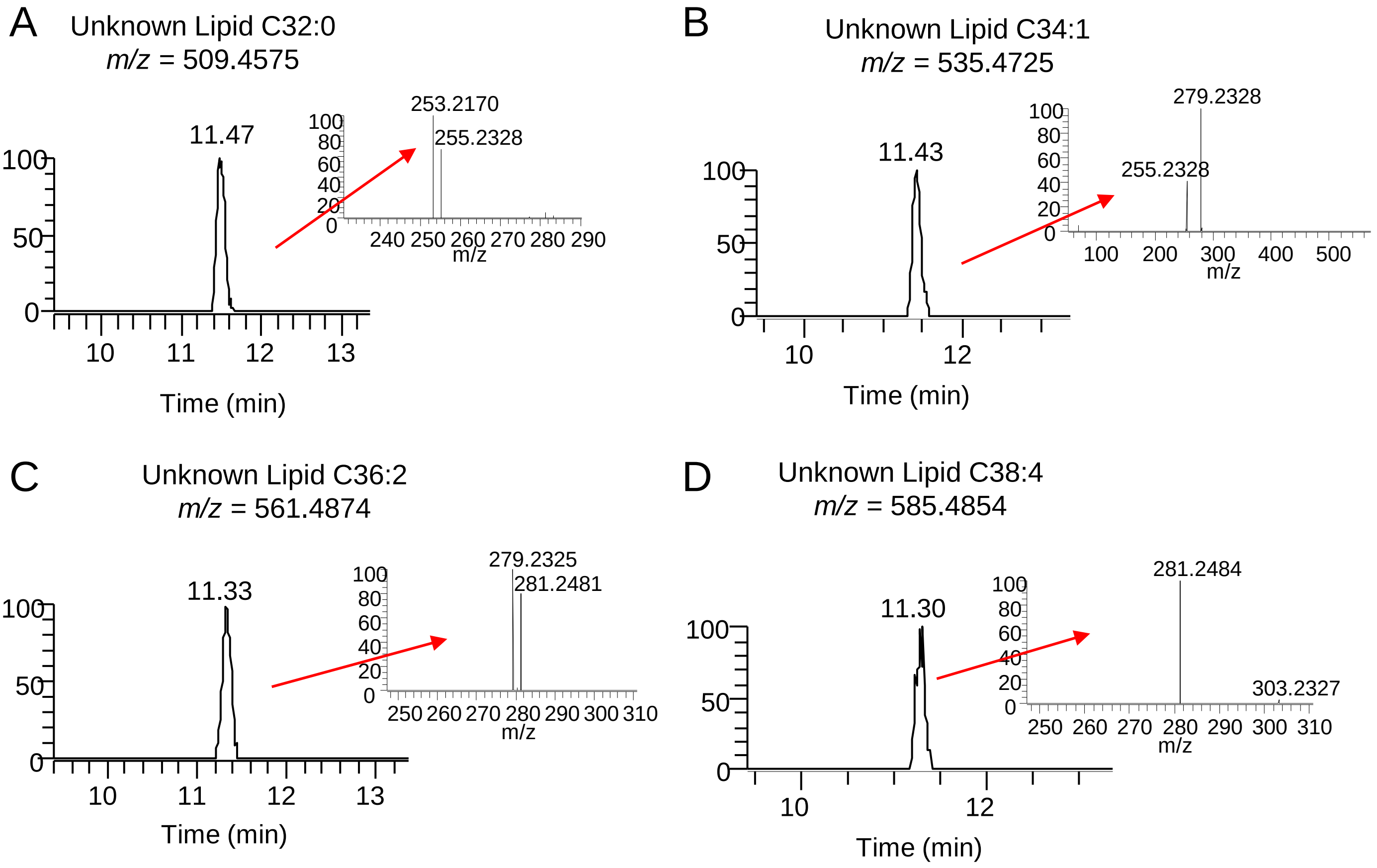

Supplemental Figure 1. (A-D) Representative extracted ion chromatograms (XIC) of unknown *m/z* lipid signal detected in Full MS mode (± 5 ppm) and their MS/MS spectra.

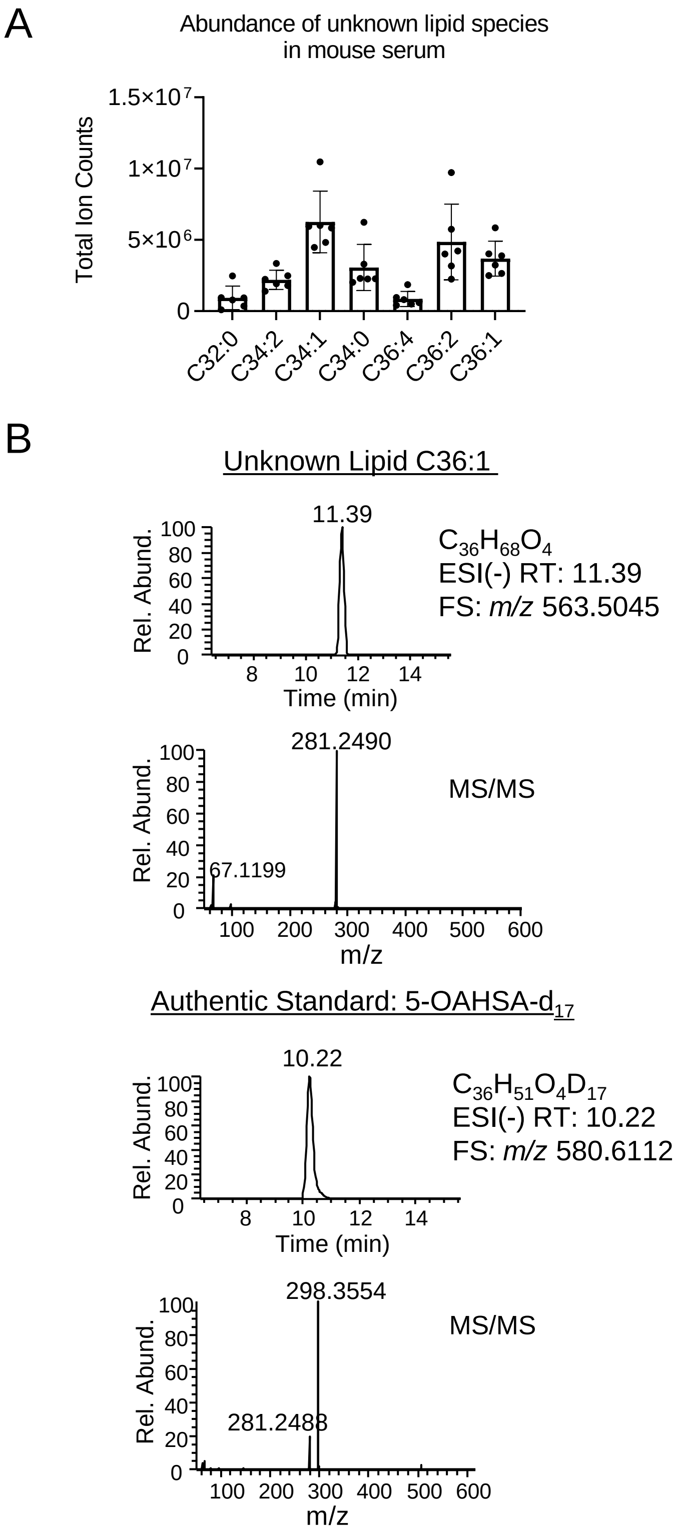

Supplemental Figure 2. Validation of unknown lipid species. (A) Detected abundance of unknown lipid species in mouse serum after a 6h fast. **(B)** XIC (±5 ppm) and MS/MS spectra of putative OAHSA signal and authentic 5-OAHSA-d_{17}.

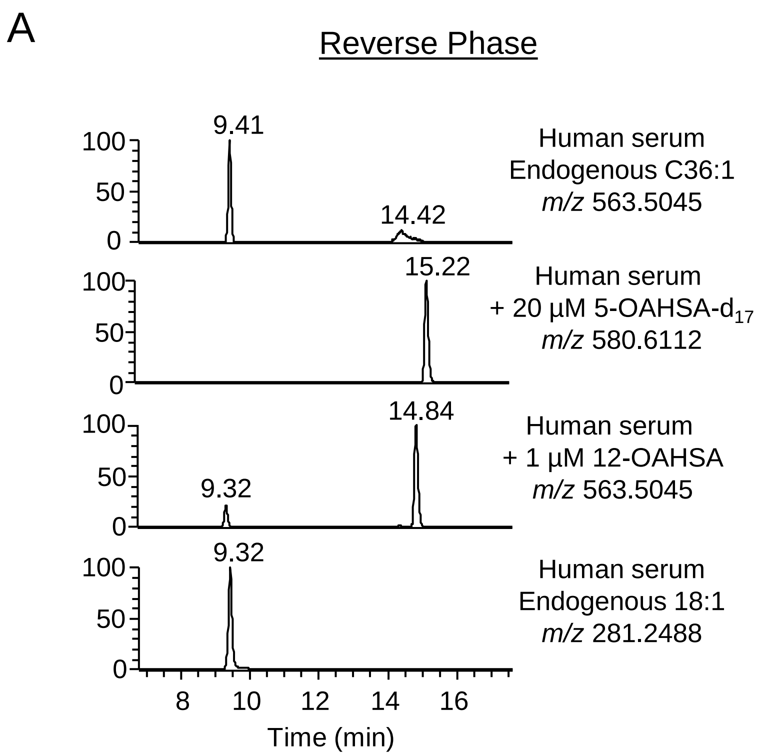

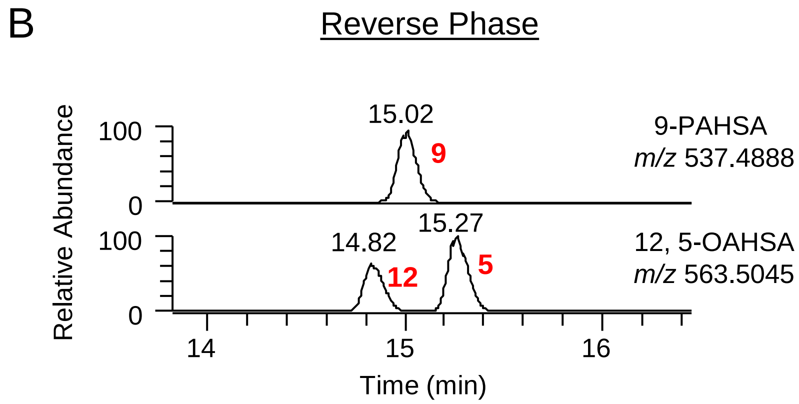

Supplemental Figure 3. Detected lipid signals on reverse phase chromatography. (A) XICs (±5 ppm) corresponding to *m/z* = 563.5045 in human serum, *m/z* = 580.6112 in human serum spiked with 20 µM labeled 5-OAHSA-d_{17}, *m/z* = 563.5045 in human serum spiked with 1 µM exogenous 12-OAHSA or *m/z* = 281.2486 (endogenous FFA 18:1) in human serum. **(B)** XICs (±5 ppm) of authentic FAHFA regioisomers dissolved in AcN/H2O. Red numbers denote regioisomer.
